# Large-Scale Candidate Gene Analysis of HDL Particle Features

**DOI:** 10.1371/journal.pone.0014529

**Published:** 2011-01-21

**Authors:** Bernhard M. Kaess, Maciej Tomaszewski, Peter S. Braund, Klaus Stark, Suzanne Rafelt, Marcus Fischer, Robert Hardwick, Christopher P. Nelson, Radoslaw Debiec, Fritz Huber, Werner Kremer, Hans Robert Kalbitzer, Lynda M. Rose, Daniel I. Chasman, Jemma Hopewell, Robert Clarke, Paul R. Burton, Martin D. Tobin, Christian Hengstenberg, Nilesh J. Samani

**Affiliations:** 1 Department of Cardiovascular Science, University of Leicester, Leicester, United Kingdom; 2 Klinik und Poliklinik für Innere Medizin II, University of Regensburg, Regensburg, Germany; 3 LipoFIT Analytic GmbH, Regensburg, Germany; 4 Institut für Biophysik und physikalische Biochemie, University of Regensburg, Regensburg, Germany; 5 Department of Preventive Medicine, Brigham and Women's Hospital, Boston, Massachusetts, United States of America; 6 Clinical Trial Service Unit, University of Oxford, Oxford, United Kingdom; 7 Deptartment of Health Sciences, University of Leicester, Leicester, United Kingdom; Leiden University Medical Center, Netherlands

## Abstract

**Background:**

HDL cholesterol (HDL-C) is an established marker of cardiovascular risk with significant genetic determination. However, HDL particles are not homogenous, and refined HDL phenotyping may improve insight into regulation of HDL metabolism. We therefore assessed HDL particles by NMR spectroscopy and conducted a large-scale candidate gene association analysis.

**Methodology/Principal Findings:**

We measured plasma HDL-C and determined mean HDL particle size and particle number by NMR spectroscopy in 2024 individuals from 512 British Caucasian families. Genotypes were 49,094 SNPs in >2,100 cardiometabolic candidate genes/loci as represented on the HumanCVD BeadChip version 2. False discovery rates (FDR) were calculated to account for multiple testing. Analyses on classical HDL-C revealed significant associations (FDR<0.05) only for CETP (cholesteryl ester transfer protein; lead SNP rs3764261: p = 5.6*10^−15^) and SGCD (sarcoglycan delta; rs6877118: p = 8.6*10^−6^). In contrast, analysis with HDL mean particle size yielded additional associations in LIPC (hepatic lipase; rs261332: p = 6.1*10^−9^), PLTP (phospholipid transfer protein, rs4810479: p = 1.7*10^−8^) and FBLN5 (fibulin-5; rs2246416: p = 6.2*10^−6^). The associations of SGCD and Fibulin-5 with HDL particle size could not be replicated in PROCARDIS (n = 3,078) and/or the Women's Genome Health Study (n = 23,170).

**Conclusions:**

We show that refined HDL phenotyping by NMR spectroscopy can detect known genes of HDL metabolism better than analyses on HDL-C.

## Introduction

HDL cholesterol (HDL-C) is an established marker of cardiovascular risk. It reflects reverse cholesterol transport (RCT) and higher plasma HDL-C is associated with lower cardiovascular risk. However, the cholesterol content of HDL particles is mainly a historically founded and analytically feasible surrogate of RCT and is it not clear whether HDL-C is indeed the best RCT-related cardiovascular risk marker [1], [2], [3], [4], [5]. HDL particles are not a homogenous class but can be further divided into subclasses. Most studies suggest that large HDL particles are associated with a favourable outcome, while small HDL particles may even be positively correlated with CV risk [1], [5], [6]. Correspondingly, the lipid disturbances of the metabolic syndrome include a decreased mean HDL particle size [3], [7]. Although HDL subclass measurements have been widely used for scientific purposes, practical clinical application is not well established yet, and there is an ongoing debate on whether lipid subclass measurements should be implemented into clinical routine [4], [5], [8].

The regulation of HDL metabolism and hence HDL particle features is not completely understood, but a strong genetic influence has been shown [9], [10]. Recently, genome-wide association (GWA) studies have identified several loci that affect HDL-C [11], [12], [13]. However, only a small proportion of the heritability of HDL-C is explained by these loci. We hypothesized that a genetic analysis of distinct HDL particle traits may provide greater sensitivity and broaden our understanding of the genetic regulation of HDL metabolism. Accordingly, we conducted a large-scale candidate gene analysis on HDL particle traits. We genotyped 49,094 single nucleotide polymorphisms (SNPs) in >2,100 cardiometabolic candidate genes and assessed mean HDL particle size and HDL particle number by NMR spectroscopy.

## Methods

### Ethics Statement

The study was approved by the Leicestershire Research Ethics Committee, and all subjects provided written informed consent. The study conforms with the principles outlined in the Declaration of Helsinki, and all procedures followed were in accordance with institutional guidelines.

### Subjects

Analyses were carried out in the GRAPHIC (Genetic Regulation of Arterial Pressure of Humans in the Community) cohort that has been previously described in detail [14]. In short, GRAPHIC contains 2,037 white European subjects in 520 nuclear families from the general population. Families were recruited by writing to women aged 40 to 69 registered with participating family practitioners in Leicestershire, UK, inviting them and their family to take part. Families were included if both parents aged 40 to 60 years and 2 offspring >18 years wished to participate. Study subjects had a detailed history taken and were examined by research nurses following standard operating procedures. Measurements included height, weight, waist-hip ratio and clinic and ambulatory blood pressure. Non-fasting blood samples were obtained for laboratory analysis.

### Lipid Phenotyping

HDL-C was determined enzymatically using the standard CHOD/PAP assay in an Olympus AU5430 analyser. GRAPHIC blood samples for NMR spectroscopy were stored at –80°C and kept unthawed until the day of NMR measurements. HDL particle size and number were determined by NMR spectroscopy at LipoFIT Analytic GmbH (Regensburg, Germany) as previously described [9]. In short, gradient weighted NMR spectra of blood plasma were recorded, which lead to characteristic overall profiles of the lipoprotein signals. Using the gradient weighted spectra, spectral regions ranging from 1.5 to 0.7 ppm were modeled into a set of 15 lipoprotein subclasses, in particular 4 HDL subclasses. Mean HDL particle size and HDL particle number were then computed from the distribution of the 4 HDL subclasses. For validation we also reconstructed HDL-C from NMR data.

### Genotyping

Genotyping was carried out using the HumanCVD BeadChip version 2 (Illumina Inc, CA) [15] based on the Infinium II genotyping platform (Illumina). Briefly, the HumanCVD BeadChip contains 49,094 SNPs in >2,100 cardiovascular candidate genes. The selection process of candidate genes and SNPs was led by investigators from the Institute of Translational Medicine and Therapeutics (ITMAT) of the University of Pennsylvania, the Broad Institute and by the National Heart Lung Institute (NHBLI) supported Candidate-gene Association resource (CARe). Genes were prioritized in three groups: [1] genes and loci with a high likelihood of functional cardiovascular significance were captured with an r^2^≥0.8 for HapMap SNPs with MAF>0.02 (Tier 1). [2] Loci that are potentially involved in cardiovascular phenotypes were covered with r^2^≥0.5 for MAF >0.05 (Tier 2). [3] For mainly larger genes of lower interest only non-synonymous and known functional variants with MAF >0.01 were captured (Tier 3). Additionally, the array contains SNPs informative of ancestry or of copy number variations as well as duplicates for quality control.

### Statistical analyses

Genotypes were carefully checked before association analyses. We excluded SNPs that represent ancestry or copy number variations, sex chromosomal SNPs and duplicates. We then applied the following filters for exclusion: missingness rate >10% (reflecting poor genotyping quality); deviation from Hardy-Weinberg equilibrium (p<0.0001) in the parental generation; minor allele frequency <0.01; and >10 Mendelian inconsistencies (also reflecting poor genotyping quality given the known familial structure in GRAPHIC).

Heritabilities of HDL-C, HDL particle size and particle number were estimated using the Sequential Oligogenic Linkage Analysis Routines (SOLAR v2.0) software[16]. In brief, total phenotypic variance of each trait was partitioned into genetic and environmental components. The mixed model applied to the data incorporated fixed effects for known covariates (age, age^2^ and sex), and variant components were calculated for genetic and residual environmental effects. The estimates were modelled to best fit the observed data using maximum likelihood approach. The total genetic component was partitioned into polygenic additive effect (narrow sense heritability) and non-additive (dominant) allelic effects.

Association analyses were carried out using generalized estimating equations (GEE) with an exchangeable correlation structure to account for the shared genetic background in families. All analyses were adjusted for gender, age and age^2^ (to account for the two generation structure of GRAPHIC). Calculations were performed in STATA (STATA Corp., Texas). Manhattan and LD plots were constructed using the *Haploview* program [17], gene centered association plots were drawn using the *Locusview* software (T. Petryshen, A. Kirby, M. Ainscow, available at www.broadinstitute.org/science/programs/medical-and-population-genetics/locusview20).

To address the problem of multiple testing we calculated false discovery rates (FDR). FDR describes the number of expected false positives among all positive findings at a certain p-value threshold [18]. We accepted an FDR of ≤0.05 as experiment-wide significant. FDR (expressed as q-values) were calculated using the R-based *q-value* tool applying the bootstrap algorithm [19].

### Replication analyses

In silico replication analyses were carried out in 23,170 women (mean age 54.2 years) of the Women Genome Health Study (WGHS) and 3,078 individuals (69.8% males and mean age 62.7 years) of the PROCARDIS cohort. The studies have been previously described in detail [20], [21]. In both studies, NMR measurements were performed using the Lipoprofile-II assay (Liposcience, Raleigh, NC). Genotypes in WGHS were imputed from GWA data based on the HumanHap300 Duo "+" chips or a combination of the HumanHuman300 Duo and iSelect chips (Illumina, San Diego, CA) with the Infinium II protocol [20]. In PROCARDIS genotypes where based on the HumanCVD bead chip (Illumina, San Diego, CA) [21].

## Results

### Subjects

Genotypes, plasma HDL-C and NMR HDL particle traits were available on 2024 GRAPHIC subjects. The characteristics of these subjects, partitioned by generation and gender are shown in **Table 1**. The values are typical of a sample of the general population in the UK indicating the representativeness of the GRAPHIC subjects. A few subjects (37) were taking lipid lowering therapy (mainly statins).

**Table 1:** Relevant characteristics of the GRAPHIC subjects partitioned by generation and gender.

	Fathers	Mothers	Sons	Daughters
Variable	(n = 512)	(n = 512)	(n = 509)	(n = 491)
Age (years)	53.8 (4.3)	51.8 (4.4)	25.0 (5.1)	25.93 (5.4)
Body mass index (kg/m2)	27.8 (4.0)	27.1 (4.5)	24.9 (4.1)	24.6 (5.0)
Mean 24-hour SBP (mmHg)	124.3 (11.5)	117.0 (11.5)	120.8 (8.1)	112.8 (7.2)
Mean 24-hour DBP (mmHg)	77.7 (7.2)	71.7 (7.6)	69.2 (6.5)	67.9 (5.2)
Total Cholesterol (mmol/L)	5.58 (0.99)	5.68 (0.98)	4.52 (0.89)	4.50 (0.82)
LDL Cholesterol (mmol/L)	3.25 (0.7)	3.26 (2.52)	2.52 (0.68)	2.50 (0.59)
HDL Cholesterol (mmol/L)	1.30 (0.3)	1.62 (0.38)	1.31 (0.27)	1.47 (0.34)
HDL particle number (nmol/L)	25,805 (3568)	25,688 (3341)	25,885 (3664)	25,752 (3497)
Mean HDL particle size (nm)	8.19 (0.25)	8.19 (0.24)	8.19 (0.25)	8.20 (0.26)
Lipid lowering therapy n (%)	25 (4.9)	11 (2.2)	0	1 (0.2%)

Data are means and standard deviations or counts and percentages.

### HDL traits

The distributions of HDL particle size and HDL particle number in the GRAPHIC subjects are shown in **Figures S1 and S2**. Both traits showed nearly normal distribution. Correlation between (enzymatically determined) HDL-C and mean HDL particle size was 0.67, between HDL-C and HDL particle number 0.41; no correlation was present between HDL particle size and HDL particle number. For validation we also reconstructed HDL-C from NMR data. These computed HDL-C values showed good agreement with enzymatically determined HDL-C (**Figure S3**). Narrow-sense heritability estimates gave values of 0.52±0.07 (r^2^±SE; p = 3.2*10^−41^), 0.35±0.04 (p = 8.0*10^−20^) and 0.44±0.04 (p = 10^−26^) for HDL-C, HDL particle number and mean HDL particle size, respectively.

### Genetic analysis

Of the 49,094 SNPs represented on the HumanCVD chip, 1,775 were excluded because they belonged to admixture and ancestry informative control markers (AIMs) and 106 because they were either duplicate SNPs or copy number variants. 12,443 SNPs were removed as their minor allele frequency was <0.01, 20 SNPs due to Mendelian errors, 424 - due to low genotyping call rate and 107 SNPs because of Hardy-Weinberg equilibrium violation. Finally, we excluded 638 SNPs on sex chromosomes and 4 SNPs due to lack of unambiguous rs identification number, leaving 33,577 SNPs for the association analyses.

**Figure 1:**
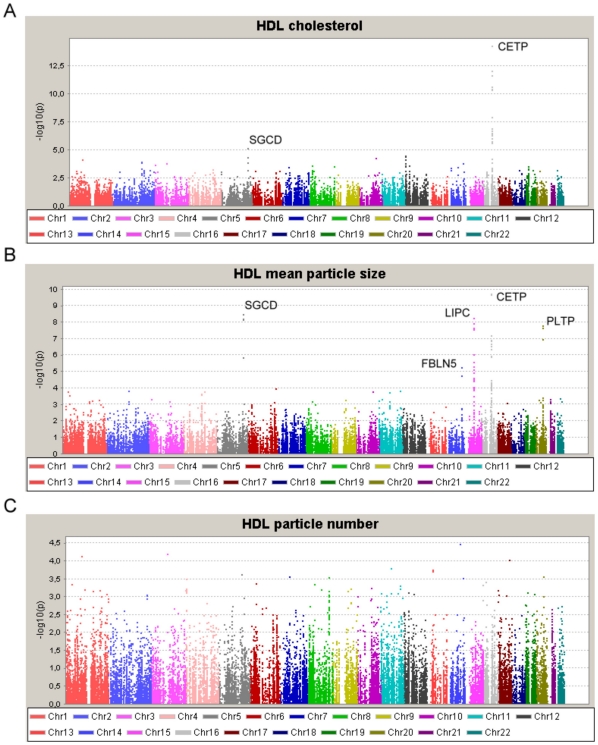
Summary of association results for HDL-C, HDL particle size and HDL particle number. Data are –log transformed p-values from association analyses for all SNPs plotted against their chromosomal location, for enzymatically determined HDL-C (panel A), NMR-derived mean HDL particle size (panel B) and NMR-derived HDL particle number (panel C). All signals with experiment-wide significance are labelled.

**Figure 2:**
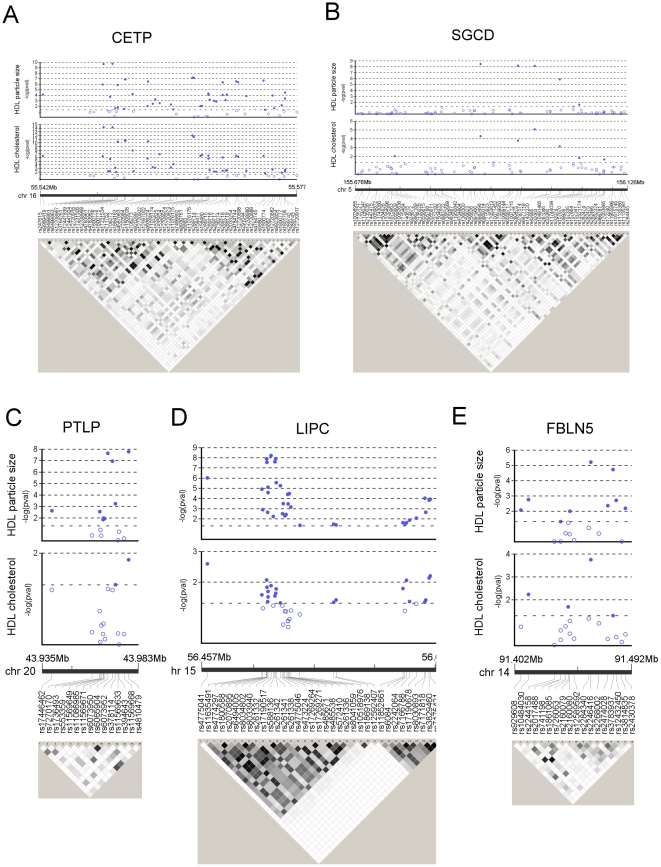
Association signals and LD structure of loci associated with HDL traits. Panel A: CETP (cholesterol ester transfer protein) gene; panel B: SGCD (sarcoglycan delta) gene; panel C: PLTP (phospholipid transer protein) gene; panel D: LIPC (hepatic lipase) gene; panel E: FBLN5 (fibulin-5) gene. In each panel, data in the upper part are –log transformed p-values from association analyses with HDL mean particle size and HDL cholesterol, adjusted for age, age^2^ and gender (open circles: p>0.01, solid circles: p≤0.01; pval – p-value). The lower part shows linkage disequilibrium (LD) structure of genotyped SNPs in the GRAPHIC cohort (r^2^-based grey shading; white: no LD (r^2^ = 0), black: complete LD (r^2^ = 1)). For legibility, in panel D (LIPC) only SNPs with significant association with mean HDL particle size (p<0.05) were plotted.

For the purpose of validation and comparison with recent GWA studies, we first examined association of the SNPs with HDL-C as routinely assessed by enzymatic assay. The genomic control coefficient (λ) showed no evidence for inflation for HDL-C (λ = 0.966). The distribution of association signals for HDL-C is shown in **Figure 1A**, and lead SNPs for loci showing experiment-wide significance (i.e. FDR q≤0.05) in **Table 2**. As expected from physiology and consistent with recent GWA studies [12], the CETP gene (encoding cholesteryl ester transfer protein) showed the strongest signal (lead SNP rs3764261, p = 5.6*10^−15^, q = 9.7*10^−11^). A significant association was also found for the SGCD gene encoding sarcoglycan delta (lead SNP rs6877118, p = 8.6*10^−6^, q = 0.012). More detailed descriptions of the association signals at the CETP and SGCD loci with HDL-C are shown in **Figure 2** **A/B**. SNPs in several other genes showed significant P values fo HDL-C; lead SNPs for the top 10 genes are shown in **Table S1**. However, besides CETP and SGCD no other gene reached experiment-wide significance for HDL-C, taking into account the number of SNPs tested.

**Table 2:** Significant association results for HDL cholesterol and HDL particle size.

HDL cholesterol								
lead SNP	Gene	Transcript	chr.	major/minor allele	MAF	beta ± SE (mmol/l)	P-value	q-value
rs3764261	CETP	cholesteryl ester transfer protein	16	G/T	0.32	0.09±0.01	5.63*10^−15^	9.7*10^−11^
rs6877118	SGCD	sarcoglycan delta	5	G/A	0.01	−0.12±0.03	8.59*10^−6^	0.012

mean HDL particle size								
lead SNP	gene	Transcript	chr.	major/minor allele	MAF	beta ± SE (nm)	p-value	q-value
rs17231506	CETP	cholesteryl ester transfer protein	16	C/T	0.32	0.051±0.008	1.94*10^−10^	3.7*10^−6^
rs6869314	SGCD	sarcoglycan delta	5	T/G	0.01	−0.13±0.021	3.51*10^−9^	3.8*10^−5^
rs261332	LIPC	hepatic lipase	15	G/A	0.20	0.059±0.010	6.13*10^−9^	3.9*10^−5^
rs4810479	PLTP	phospholipid transfer protein	20	T/C	0.27	−0.048±0.009	1.67*10^−8^	6.0*10^−5^
rs2246416	FBLN5	fibulin-5	14	A/G	0.29	0.040±0.009	6.22 * 10^−6^	0.0070

Lead SNPs of genes with experiment-wide significant (q<0.05) association tests. Results are from GEE regression analyses adjusted for age, age^2^ and gender. SNP, single nucleotide polymorpism; chr., chromosome; MAF, minor allele frequency in the parental generation; Beta, beta coefficient per minor allele copy; SE; standard error. A negative beta coefficient indicates a lower value for the trait for each copy of the minor allele.

We next examined the associations with mean HDL particle size (**Figure 1B** **,** **Table 2** **and Table S2**) as retrieved from NMR spectroscopy. Genomic inflation factor λ was 0.949 for HDL particle size. Again polymorphisms in the CETP gene showed the most significant association (lead SNP rs17231506, p = 1.9*10^−10^, q = 3.7*10^−6^) (**Figure 2A**). The second strongest association was again found for SGCD (lead SNP rs10071215, p = 3.5×10^−9^, q = 3.8*10^−5^; **Figures 1B** **and** **2B**). The lowest p-value for HDL particle size in both these genes was seen with a different SNP to that for HDL-C (**Table 2**). However, in both instances the relevant SNPs were in strong LD with each other (**Figures** **2A and 2B**), suggesting that these are mapping the same signals. Besides CETP and SGCD, association analysis of HDL particle size revealed experiment-wide significant associations in three further genes (**Figure 1B**): LIPC (encoding hepatic lipase, lead SNP rs261332, p = 6.1*10^−9^, q = 3.9*10^−5^), PLTP (encoding phospholipid transfer protein, lead SNP rs4810479: p = 1.7*10^−8^, q = 6.0*10^−5^) and FBLN5 (encoding fibulin-5, lead SNP rs2246416, p = 6.2*10^−6^, q = 0.007) (**Table 2**). More detailed information of the location of the association signals in these genes and the LD between SNPs are shown in **Figures 2C, 2D and 2E**, respectively.

The association analysis with NMR-derived HDL particle number did not yield experiment-wide significant results (**Figure 1C**). The ten genes with the strongest association results for particle number are shown in **Table S3**.

We next analysed two top SNPs for each of our two novel suggestive loci (SGCD: rs10071215 and rs6877118; Fibulin-5: rs2246416 and rs3783937), in two independent cohorts: the Women's Genome Health Study (WGHS) and the PROCARDIS cohort [20], [21]. In WGHS, the two SGCD SNPs were not imputable due to low minor allele frequency. In PROCARDIS the two SCGD SNPs were directly genotyped and showed no association with mean HDL particle size (**Table S4**). The two Fibulin-5 SNPs were not associated with HDL particle size in either WGHS or PROCARDIS (**Table S4**).

## Discussion

We measured HDL subclass distribution by NMR spectroscopy in >500 families of the GRAPHIC cohort. The calculated heritability of mean HDL particle size (∼44%) in the GRAPHIC study is in line with the literature (∼28–52%), while that of HDL particle number (∼35%) is somewhat lower than previously reported (48–65%) [9], [22]. Conducting a large-scale candidate gene association analysis with mean HDL particle size and HDL particle number, we found that the analysis of HDL particle size detected genes known to be involved in HDL metabolism better than the equally powered scan on enzymatically determined HDL-C. We found significant association of Fibulin-5 and SGCD polymorphisms with HDL particle size, but were not able to replicate these findings in additional cohorts.

From a mechanistic point of view, HDL cholesterol level is determined by two factors: first the number of circulating HDL particles, and second the mean cholesterol content per particle, i.e. its size or subclass distribution. It is likely that these two variables are regulated by distinct biological mechanisms. Given the increasingly compelling evidence that cardiovascular risk related to HDL-C is mediated by specific features of HDL particles, understanding the genetic regulation of these features and also which features are affected by loci associated with HDL-C has important clinical relevance. HDL subclasses and particle number can be assessed by several techniques. Ultracentrifugation, gel electrophoresis, precipitation and immunological assays are established methods to separate HDL particles into subfractions. More recently, NMR spectroscopy has become increasingly used for determining lipid subfractions [5], [9], [23].

The proof of principle for our hypothesis that refined HDL phenotyping would provide increased sensitivity in detecting loci affecting HDL metabolism compared with HDL-C is illustrated by the findings for PLTP and LIPC. Both proteins are associated with HDL metabolism. PLTP transfers phospholipids from triglyceride-rich lipoproteins (VLDL) to HDL particles while hepatic lipase hydrolyses triglycerides and phospholipids and also facilitates receptor binding of lipoprotein particles [24]. These functions may explain the stronger association of variants observed in these genes with HDL particle size (which will be influenced by their triglyceride/phospholipid content) than with HDL cholesterol. Furthermore, although the C allele of lead SNP rs4810479 is associated with an increased mean particle size, it is at the same time associated with decreased particle number albeit not at an experiment-wide level of significance (p = 0.002; q = 0.71). As a result, the association with HDL cholesterol is markedly weakened (p = 0.014) (**Table S2**). While variants in both genes have now been associated with HDL-C, it is notable that for PLTP a meta-analysis of over 19.000 individuals was required for the signal to reach genome-wide significance [12]. This again demonstrates the advantages of distinct HDL particle phenotyping. During the preparation of this manuscript, Chasman *et al.* published a large genome-wide association study for NMR-based lipid traits [20], and found similar associations between HDL particle size and polymorphisms in CETP, LIPC and PLTP. The totality of the evidence for these loci therefore indicates that their effect on HDL-C is primarily mediated by their impact on HDL particle size.

We observed potentially novel associations with HDL particle size of variants in the sarcoglycan delta coding gene (SGCD) and in the fibulin-5 gene (FBLN5). The signal in SCGD is based on four SNPs (rs6869314, rs10071215, rs6877118, rs10491465) which all have very low minor allele frequencies (0.010–0.014) and are in high LD (pairwise r^2^ 0.6-0.9). SCGD protein has been associated with development of cardiomyopathies [25]. There is no pre-existing evidence for its involvement in lipid metabolism. Given the low allele frequencies, the signal is based on rougly 40 heterozygous individuals. Given that no association was seen in PROCARDIS, we have to conclude that this finding is likely to be false positive.

In contrast to the association in SCGD, that in FBLN5 was based on 798 heterozygous and 176 homozygous individuals for the minor allele. Furthermore, of 18 SNPs analysed in the FBLN5 gene, 9 show a nominal significant association with HDL particle size (**Figure 2E**). Fibulin-5 (syn. ARMD3, DANCE) is an extracellular matrix protein that has been implicated in vascular development and remodelling [26]. It is required for the assembly and organization of elastic fibers [27] (DANCE: developmental arteries and neural crest EGF-like protein). Mutations have been associated with recessive cutis laxa and age-related macular degeneration (ARMD) [28], [29]. The association of FBLN5 with ARMD is particularly interesting, because this condition is defined by the deposition of cholesterol containing drusen, an obvious parallel with atherosclerotic lesions [30]. ARMD has been consistently associated with apoE polymorphisms and HDL particle traits, providing further evidence for a role of reverse cholesterol transport in this condition [31], [32]. Moreover, fibulin-5 is upregulated in atherosclerotic and ballon-injured vessel wall [33]. Hence, one possible explanation of the association between FBLN5 polymorphisms and HDL particle size is the affection of cholesterol flux to HDL particles in the vessel wall. Fibulin-5 has furthermore been shown to bind superoxide dismutase (SOD) [34]. As oxidative stress affects HDL subclass distribution [35], an alternative explanation would be an effect of FBLN5 polymorphisms on SOD activity in the vessel wall and hence an altered oxidative state. However, we were not able to replicate association between Fibulin-5 polymorphisms and HDL particle size in two independent cohorts, and hence the significance of our finding in GRAPHIC remains doubtful.

Interestingly, we did not observe any experiment-wide significant associations with HDL particle number. The negative finding may be explained by the fact that HDL particle number has a somewhat weaker heritability (h^2^ = 0.35) than HDL cholesterol and mean HDL particle size (h^2^ = 0.52 and h^2^ = 0.44, respectively). Another possible explanation is that the genes regulating particle number may not be represented on the HumanCVD BeadChip. This possibility is supported by the fact that the HumanCVD BeadChip chip contains candidate genes mainly based on classical cardiometabolic traits. However, HDL cholesterol is much more determined by mean cholesterol content per particle than by particle number. In our data, NMR-derived HDL mean particle size explains 45% of the variance of enzymatically determined HDL cholesterol, while HDL particle number accounts for only 17% of HDL-C variance. Hence, genes controlling HDL particle number are difficult to discover using traditional lipid phenotyping. Therefore, genes affecting HDL particle number may be underrepresented on the HumanCVD BeadChip. However, at least two out of the top ten loci where we found associations with HDL particle number (**Table S3**) have previously been implicated in lipid traits: HNF4a (encoding hepatocyte nuclear factor α; lead SNP rs3212197, p = 0.00028, q = 0.64) is a known MODY (mature onset diabetes of the young) gene that has been associated with HDL cholesterol and apoA1 in recent GWAS [20], [36], while TRIB1 (tribbles homolog 1; lead SNP rs4871598, p = 0.00030, q = 0.64) has been correlated with triglyceride levels in the same studies.

### Limitations

Some limitations of our study should be noted. First, although large-scale, our study was nonetheless a candidate gene analysis. The HumanCVD BeadChip array specifically targets known genes/loci involved or suspected to be involved in HDL metabolism including those from available GWAS studies, but is limited in its ability to discover hitherto completely unsuspected genes. Also, our blood samples were taken from non-fasting individuals which could impact on lipid measurements. However, recent data suggest that the differences between fasting and non-fasting state are small with respect to HDL particle features [20].

Given that meta-analyses of several tens of thousands of subjects have been required to robustly detect some of the genetic variants affecting HDL cholesterol, an important limitation of the study is power. This together with the limited sampling of the genome undoubtedly means that many loci that affect HDL-C and HDL particle features have not been detected. However, our principle objective was to compare genetic association signals seen for HDL-C and that for HDL particle features in the same population and obtain some novel mechanistic insights related to some of the associations affecting HDL metabolism. In this regard, the nuclear family study design limited the impact of any population structure and the fact that the GRAPHIC cohort is representative of the general European Caucasian population means that our findings are generalisable, at least to this population.

### Conclusion

We present a large-scale candidate gene analyses on NMR-derived HDL particle traits in over 2,000 individuals. We show that refined HDL phenotyping can detect known genes of HDL metabolism better than analyses on enzymatically determined HDL-C and may broaden understanding of HDL biology.

## Supporting Information

Figure S1 Distribution of mean HDL particle size in the GRAPHIC cohort. Brown bars: Density histogram of mean HDL particle size in the GRAPHIC cohort. Continuous line: normal distribution.
(0.60 MB TIF)

Figure S2 Distribution of HDL particle number in the GRAPHIC cohort. Brown bars: Density histogram of mean HDL particle size in the GRAPHIC cohort. Continuous line: normal distribution.
(0.64 MB TIF)

Figure S3 Correlation of HDL cholesterol determined enzymatically with HDL cholesterol computed from NMR-derived HDL subclass data.
(0.28 MB TIF)

Table S1 Lead SNPs for 10 genes with the strongest association results for enzymatically determined HDL-C. Results are from GEE regression analyses adjusted for age, age^^2^^ and gender. The right part of the table shows association results of the SNPs with the other two measured traits' mean HDL particle size and HDL particle number. Chr.: chromosome; MAF: minor allele frequency; beta: beta coefficient per minor allele copy; SE: standard error. A negative beta coefficient indicates a lower value for the trait for each copy of the minor allele. Gene abbreviations: CETP: cholesteryl ester transfer protein; SGCD: sarcoglycan delta; CACNA1C: voltage dependent calcium channel L type alpha 1C subunit; CHUK: conserved helix-loop-helix ubiquitous kinase; TNNI3K: TNNI3 interacting kinase; ABCB11: ATP-binding cassette subfamily B member 11; SLC12A3: solute carrier family 12 member 3; FBLN5: fibulin 5; FOXP1: forkhead box P1; CAV3: caveolin 3.
(0.03 MB DOC)

Table S2 Lead SNPs for 10 genes with the strongest association results for mean HDL particle size. Results are from GEE regression analyses adjusted for age, age^^2^^ and gender. The right part of the table shows association results of the SNPs with the other two measured traits' mean HDL cholesterol and HDL particle number. Chr.: chromosome; MAF: minor allele frequency; beta: beta coefficient per minor allele copy; SE: standard error. A negative beta coefficient indicates a lower value for the trait for each copy of the minor allele. Gene abbreviations: CETP: cholesteryl ester transfer protein; SGCD: sarcoglycan delta; LIPC: hepatic lipase; PLTP: phospholipid transfer protein; FBLN5: fibulin 5; SLC22A2: solute carrier family 22 member 2; SMPD4: neutral sphingomyelinase-3; HSPA8: heat shock 70 kDa protein 8; CHUK: conserved helix-loop-helix ubiquitous kinase; TNIP3: TNFAIP3 interacting protein.
(0.03 MB DOC)

Table S3 Lead SNPs for 10 genes with the strongest association results for HDL particle number. Results are from GEE regression analyses adjusted for age, age^2^ and gender. The right part of the table shows association results of the SNPs with the other two measured traits' HDL cholesterol and mean HDL particle size. Chr.: chromosome; MAF: minor allele frequency; beta: beta coefficient per minor allele copy; SE: standard error. A negative beta coefficient indicates a lower value for the trait for each copy of the minor allele. Gene abbreviations: TGFB3: transforming growth factor beta 3; PROS1: protein S alpha; ABCA4: ATP-binding cassette subfamily A member 4; KCNJ2: potassium inwardly rectifying channel subfamily J member 2; CNTF: ciliary neurotrophic factor; STARD13: START domain containing 13; IL4: interleukin 4; EGFR: epidermal growth factor receptor; HNF4A: hepatocyte nuclear factor 4 alpha; TRIB1: tribbles homolog 1.
(0.03 MB DOC)

Table S4 Replication analyses in WGHS and PROCARDIS. SGCD SNPs in WGHS were not imputable due to low minor allele frequency. Beta-coefficients are given per copy of minor allele, assuming an additive model of inheritance, adjusted for age (and gender in PROCARDIS). rsq: imputation quality measure; MAF: minor allele frequency * Due to low MAF, homozygous and heterozygous carriers of the minor allele were pooled, hence the beta-coefficient is given for the presence of the minor allele.
(0.03 MB DOC)
