# Supplementary figures and images for: Large-Scale Candidate Gene Analysis of HDL Particle Features
Source: PLoS One. 2011 Jan 21;6(1):e14529. doi: 10.1371/journal.pone.0014529 (PMC3024972; doi:10.1371/journal.pone.0014529)

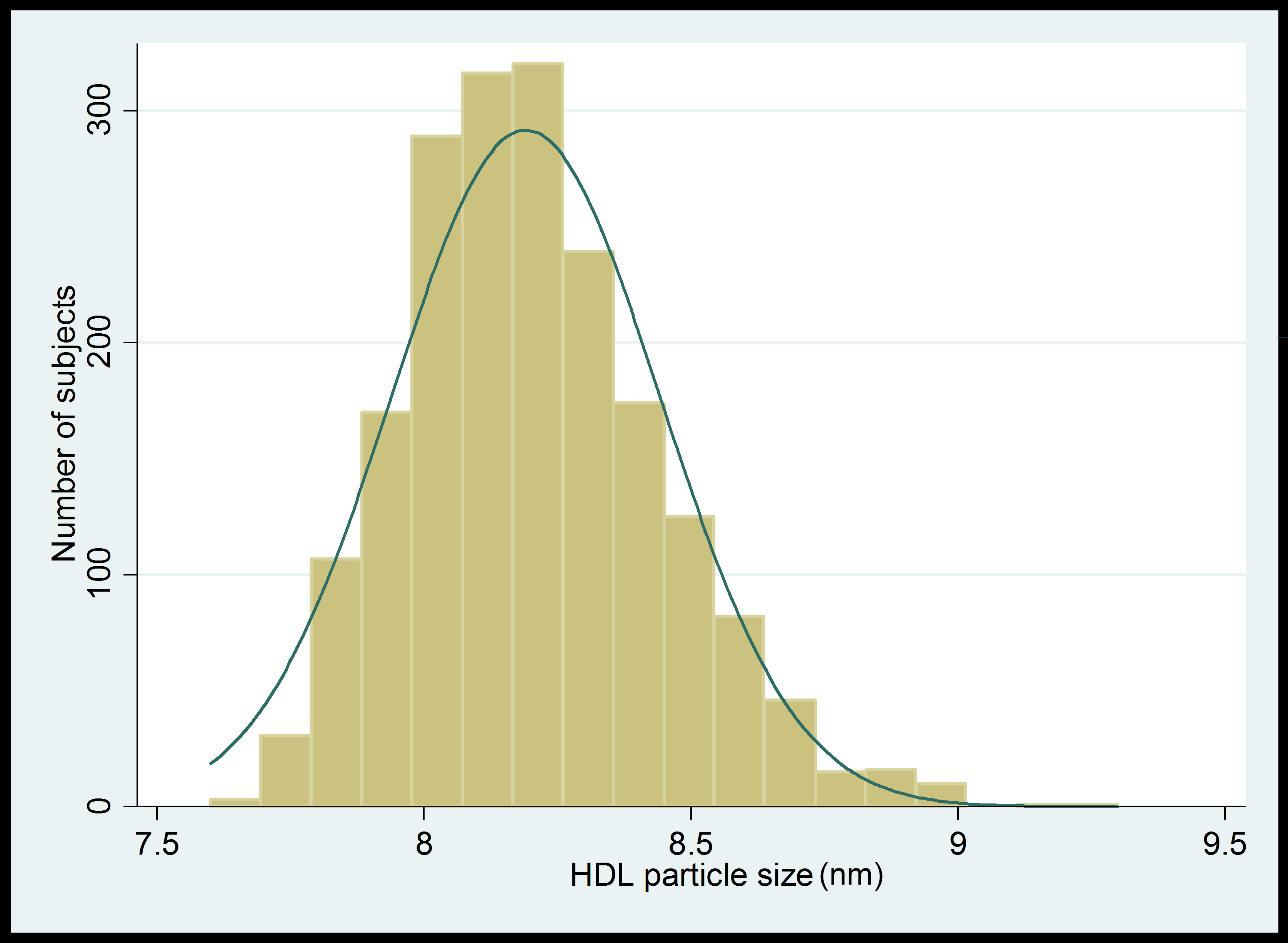

Supplement: Figure S1 — Distribution of mean HDL particle size in the GRAPHIC cohort. Brown bars: Density histogram of mean HDL particle size in the GRAPHIC cohort. Continuous line: normal distribution. (0.60 MB TIF) [file pone.0014529.s001.tif]

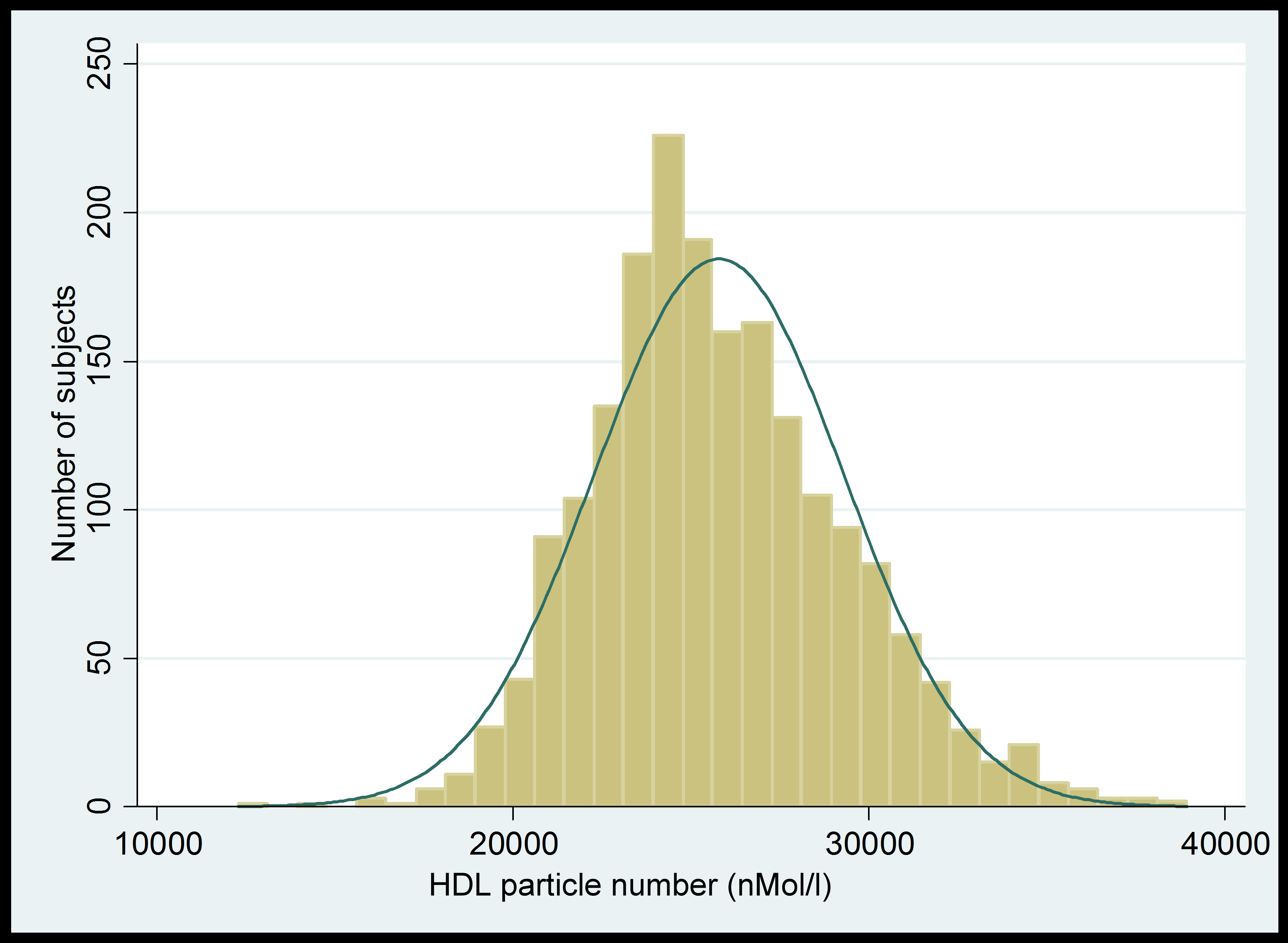

Supplement: Figure S2 — Distribution of HDL particle number in the GRAPHIC cohort. Brown bars: Density histogram of mean HDL particle size in the GRAPHIC cohort. Continuous line: normal distribution. (0.64 MB TIF) [file pone.0014529.s002.tif]

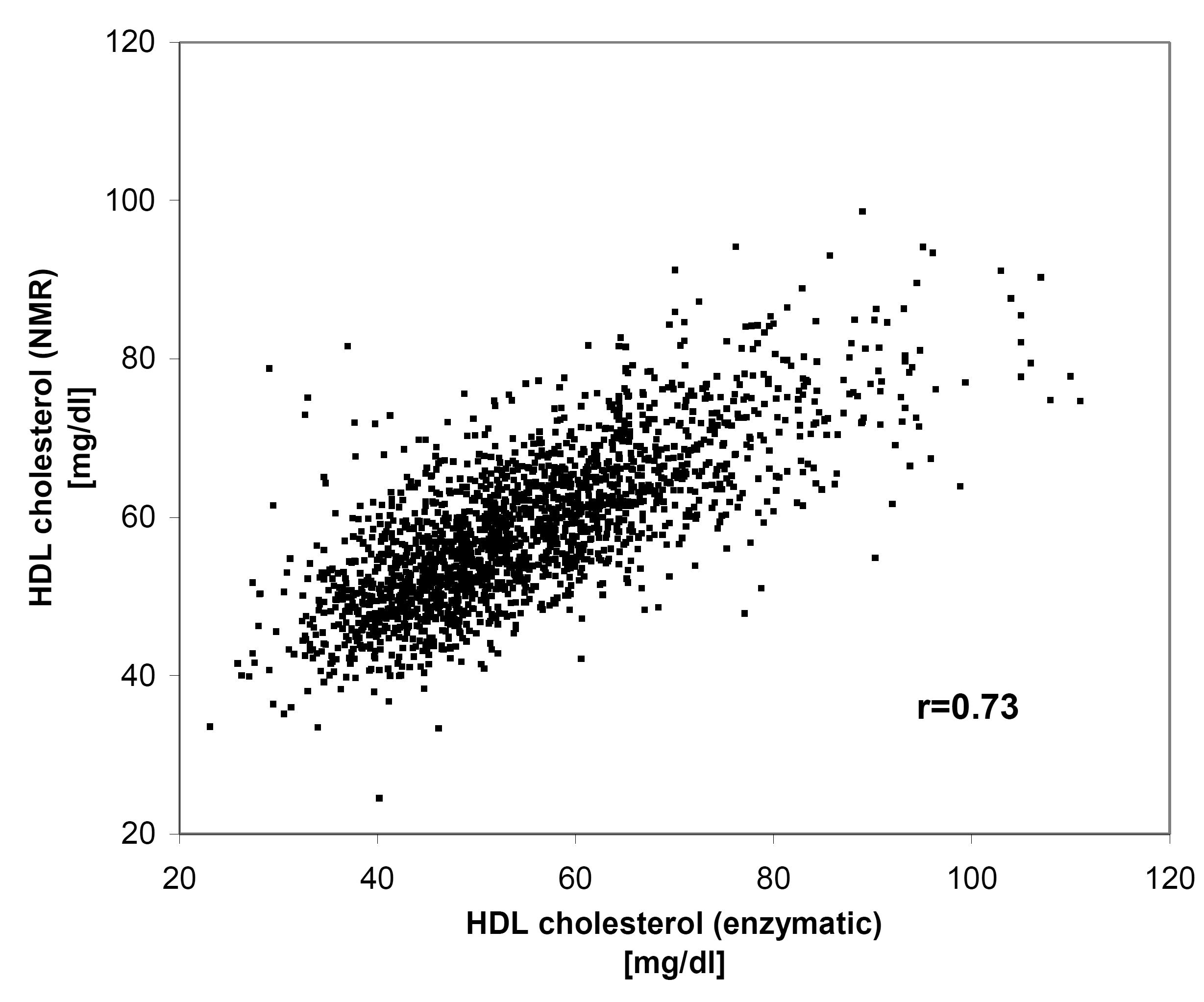

Supplement: Figure S3 — Correlation of HDL cholesterol determined enzymatically with HDL cholesterol computed from NMR-derived HDL subclass data. (0.28 MB TIF) [file pone.0014529.s003.tif]
