# Supplementary material for: Large-Scale Candidate Gene Analysis of HDL Particle Features
Source: PLoS One. 2011 Jan 21;6(1):e14529. doi: 10.1371/journal.pone.0014529 (PMC3024972; doi:10.1371/journal.pone.0014529)
Supplement: Table S1 — Lead SNPs for 10 genes with the strongest association results for enzymatically determined HDL-C. Results are from GEE regression analyses adjusted for age, age2 and gender. The right part of the table shows association results of the SNPs with the other two measured traits' mean HDL particle size and HDL particle number. Chr.: chromosome; MAF: minor allele frequency; beta: beta coefficient per minor allele copy; SE: standard error. A negative beta coefficient indicates a lower value for the trait for each copy of the minor allele. Gene abbreviations: CETP: cholesteryl ester transfer protein; SGCD: sarcoglycan delta; CACNA1C: voltage dependent calcium channel L type alpha 1C subunit; CHUK: conserved helix-loop-helix ubiquitous kinase; TNNI3K: TNNI3 interacting kinase; ABCB11: ATP-binding cassette subfamily B member 11; SLC12A3: solute carrier family 12 member 3; FBLN5: fibulin 5; FOXP1: forkhead box P1; CAV3: caveolin 3. (0.03 MB DOC) [file pone.0014529.s004.doc]

|  | | | | | | | **HDL cholesterol** | | | **HDL particle size** | | **HDL particle number** | |
| --- | --- | --- | --- | --- | --- | --- | --- | --- | --- | --- | --- | --- | --- |
| **lead SNP** | **chr.** | **position**  **(bp)** | **gene** | SNP location | major/minor allele | **MAF** | **beta ± SE**  **(mmol/l)** | **p-value** | **q-value** | **beta ± SD**  **(nm)** | **p-value** | **beta ± SE**  **(nmol/l)** | **p-value** |
| rs3764261 | 16 | 55550825 | CETP | 5’upstream | G/T | 0.32 | 0.092±0.012 | 5.6*10^-15^ | 9.7*10^-11^ | 0.051±0.008 | 2.2*10^-10^ | 34±126 | 0.79 |
| rs6877118 | 5 | 155972566 | SGCD | Intron | G/A | 0.01 | -0.120±0.027 | 8.6*10^-6^ | 0.012 | -0.118±0.020 | 7.2*10^-9^ | 379±509 | 0.46 |
| rs7295775 | 12 | 2647666 | CACNA1C | Intron | T/C | 0.05 | 0.112±0.027 | 4.1*10^-5^ | 0.055 | 0.035±0.018 | 0.056 | 786±268 | 0.0033 |
| rs17112737 | 10 | 101960811 | CHUK | Intron | G/A | 0.01 | -0.146±0.036 | 5.7*10^-5^ | 0.069 | -0.099±0.026 | 0.00017 | -209±482 | 0.67 |
| rs984167 | 1 | 74754135 | TNNI3K | Intron | T/A | 0.01 | -0.131±0.033 | 8.2*10^-5^ | 0.090 | -0.085±0.028 | 0.0022 | -833±370 | 0.024 |
| rs2544367 | 2 | 169504534 | ABCB11 | Intron | C/T | 0.46 | 0.044±0.012 | 0.00013 | 0.14 | 0.024±0.008 | 0.0032 | 32±111 | 0.77 |
| rs12446689 | 16 | 55495517 | SLC12A3 | Intron | A/G | 0.15 | -0.052±0.014 | 0.00017 | 0.16 | -0.024±0.010 | 0.019 | -202±166 | 0.22 |
| rs2246416 | 14 | 91460373 | FBLN5 | Intron | C/G | 0.29 | 0.049±0.013 | 0.00018 | 0.16 | 0.040±0.009 | 6.2*10^-6^ | 61±132 | 0.65 |
| rs11719587 | 3 | 71268320 | FOXP1 | Intron | A/T | 0.01 | -0.117±0.031 | 0.00019 | 0.16 | -0.056±0.021 | 0.0068 | -559±456 | 0.22 |
| rs237872 | 3 | 8755438 | CAV3 | Intron | C/T | 0.50 | -0.043±0.012 | 0.00025 | 0.21 | -0.020±0.008 | 0.011 | -214±108 | 0.047 |
