# Supplementary material for: Large-Scale Candidate Gene Analysis of HDL Particle Features
Source: PLoS One. 2011 Jan 21;6(1):e14529. doi: 10.1371/journal.pone.0014529 (PMC3024972; doi:10.1371/journal.pone.0014529)
Supplement: Table S2 — Lead SNPs for 10 genes with the strongest association results for mean HDL particle size. Results are from GEE regression analyses adjusted for age, age2 and gender. The right part of the table shows association results of the SNPs with the other two measured traits' mean HDL cholesterol and HDL particle number. Chr.: chromosome; MAF: minor allele frequency; beta: beta coefficient per minor allele copy; SE: standard error. A negative beta coefficient indicates a lower value for the trait for each copy of the minor allele. Gene abbreviations: CETP: cholesteryl ester transfer protein; SGCD: sarcoglycan delta; LIPC: hepatic lipase; PLTP: phospholipid transfer protein; FBLN5: fibulin 5; SLC22A2: solute carrier family 22 member 2; SMPD4: neutral sphingomyelinase-3; HSPA8: heat shock 70 kDa protein 8; CHUK: conserved helix-loop-helix ubiquitous kinase; TNIP3: TNFAIP3 interacting protein. (0.03 MB DOC) [file pone.0014529.s005.doc]

|  | | | | | | | **mean HDL particle size** | | | **HDL cholesterol** | | **HDL particle number** | |
| --- | --- | --- | --- | --- | --- | --- | --- | --- | --- | --- | --- | --- | --- |
| **lead SNP** | **chr.** | **position (bp)** | **gene** | **SNP location** | **major/minor allele** | **MAF** | **beta ± SE**  **(nm)** | **p-value** | **q-value** | **beta ± SE**  **(mmol/l)** | **p-value** | **beta ± SE**  **(nmol/l)** | **p-value** |
| rs17231506 | 16 | 55552029 | CETP | 5’upstream | C/T | 0.32 | 0.051±0.008 | 1.9 * 10^-10^ | 3.7*10^-6^ | 0.091±0.012 | 5.9*10^-15^ | 32±126 | 0.80 |
| rs6869314 | 5 | 155883274 | SGCD | Intron | T/G | 0.01 | -0.127±0.021 | 3.5 * 10^-9^ | 3.8*10^-5^ | -0.118±0.029 | 0.00005 | 518±557 | 0.35 |
| rs261332 | 15 | 56514617 | LIPC | Intron | G/A | 0.20 | 0.059±0.01 | 6.1 * 10^-9^ | 3.9*10^-5^ | 0.036±0.014 | 0.013 | -138±143 | 0.33 |
| rs4810479 | 20 | 43978455 | PLTP | 5’upstream | T/C | 0.27 | -0.048±0.008 | 1.7 * 10^-8^ | 6.0*10^-5^ | -0.030±0.012 | 0.014 | 379±123 | 0.002 |
| rs2246416 | 14 | 91460373 | FBLN5 | Intron | A/G | 0.29 | 0.040±0.009 | 6.2 * 10^-6^ | 0.0070 | 0.049±0.013 | 0.0002 | 61±132 | 0.65 |
| rs3798158 | 6 | 160566212 | SLC22A2 | Intron | A/C | 0.06 | 0.072±0.018 | 0.00012 | 0.081 | 0.090±0.027 | 0.0009 | 136±241 | 0.57 |
| rs10198499 | 2 | 130659983 | SMPD4 | Intron | T/C | 0.39 | -0.027±0.008 | 0.00016 | 0.098 | -0.008±0.011 | 0.48 | 92±113 | 0.41 |
| rs7944075 | 11 | 122438887 | HSPA8 | 5’upstream | C/T | 0.03 | -0.065±0.017 | 0.00016 | 0.098 | -0.052±0.025 | 0.040 | 370±255 | 0.30 |
| rs17112737 | 10 | 101960811 | CHUK | Intron | G/A | 0.01 | -0.099±0.026 | 0.00017 | 0.11 | -0.146±0.036 | 0.00006 | -209±482 | 0.67 |
| rs13129450 | 4 | 122310683 | TNIP3 | Unknown | T/G | 0.31 | 0.03±0.008 | 0.00018 | 0.11 | 0.016±0.012 | 0.20 | -212±128 | 0.096 |
