# Supplementary material for: Large-Scale Candidate Gene Analysis of HDL Particle Features
Source: PLoS One. 2011 Jan 21;6(1):e14529. doi: 10.1371/journal.pone.0014529 (PMC3024972; doi:10.1371/journal.pone.0014529)
Supplement: Table S3 — Lead SNPs for 10 genes with the strongest association results for HDL particle number. Results are from GEE regression analyses adjusted for age, age2 and gender. The right part of the table shows association results of the SNPs with the other two measured traits' HDL cholesterol and mean HDL particle size. Chr.: chromosome; MAF: minor allele frequency; beta: beta coefficient per minor allele copy; SE: standard error. A negative beta coefficient indicates a lower value for the trait for each copy of the minor allele. Gene abbreviations: TGFB3: transforming growth factor beta 3; PROS1: protein S alpha; ABCA4: ATP-binding cassette subfamily A member 4; KCNJ2: potassium inwardly rectifying channel subfamily J member 2; CNTF: ciliary neurotrophic factor; STARD13: START domain containing 13; IL4: interleukin 4; EGFR: epidermal growth factor receptor; HNF4A: hepatocyte nuclear factor 4 alpha; TRIB1: tribbles homolog 1. (0.03 MB DOC) [file pone.0014529.s006.doc]

|  | | | | | | | **HDL particle number** | | | **HDL cholesterol** | | **mean HDL particle size** | |
| --- | --- | --- | --- | --- | --- | --- | --- | --- | --- | --- | --- | --- | --- |
| **lead SNP** | **chr.** | **pos (bp)** | **gene** | **SNP location** | **major/**  **minor allele** | **MAF** | **beta ± SE**  **(nmol/l)** | **p-value** | **q-value** | **beta ± SE**  **(mmol/l)** | **p-value** | **beta ± SE**  **(nm)** | **p-value** |
| rs3917151 | 14 | 75515800 | TGFB3 | Intron | G/A | 0.01 | -930±224 | 3.4*10^-5^ | >0.5 | -0.029±0.039 | 0.47 | -0.0191±0.030 | 0.53 |
| rs4857037 | 3 | 95110502 | PROS1 | Intron | A/G | 0.08 | -797±200 | 6.6*10^-5^ | >0.5 | -0.028±0.023 | 0.22 | 0.0046±0.016 | 0.77 |
| rs4147799 | 1 | 94357047 | ABCA4 | Intron | T/C | 0.01 | -1651±418 | 7.7*10^-5^ | >0.5 | -0.120±0.045 | 0.0081 | 0.0052±0.031 | 0.86 |
| rs1468472 | 17 | 65682344 | KCNJ2 | untranslated | C/T | 0.04 | 983±252 | 9.5*10^-5^ | >0.5 | 0.042±0.028 | 0.13 | 0.0060±0.018 | 0.75 |
| rs1800169 | 11 | 58148077 | CNTF | Intron | G/A | 0.15 | 609±162 | 0.00017 | >0.5 | 0.006±0.016 | 0.69 | -0.0092±0.011 | 0.39 |
| rs2764615 | 13 | 32604354 | STARD13 | Intron | G/A | 0.43 | -421±113 | 0.00018 | >0.5 | -0.026±0.011 | 0.020 | -0.0037±0.008 | 0.63 |
| rs2243297 | 5 | 132027070 | IL4 | 3’ downstr. | T/A | 0.03 | -1074±293 | 0.00025 | >0.5 | 0.043±0.041 | 0.30 | 0.0424±0.027 | 0.12 |
| rs6956366 | 7 | 55068995 | EGFR | Intron | G/C | 0.33 | -411±113 | 0.00028 | >0.5 | -0.015±0.012 | 0.21 | 0.0026±0.009 | 0.76 |
| rs3212197 | 20 | 42477621 | HNF4A | Intron | C/T | 0.08 | 714±197 | 0.00028 | >0.5 | 0.031±0.020 | 0.12 | -0.0057±0.014 | 0.68 |
| rs4871598 | 8 | 126529172 | TRIB1 | 3’ UTR | G/A | 0.26 | 440±122 | 0.00030 | >0.5 | 0.011±0.013 | 0.40 | 0.0075±0.010 | 0.43 |
