# Supplementary material for: Large-Scale Candidate Gene Analysis of HDL Particle Features
Source: PLoS One. 2011 Jan 21;6(1):e14529. doi: 10.1371/journal.pone.0014529 (PMC3024972; doi:10.1371/journal.pone.0014529)
Supplement: Table S4 — Replication analyses in WGHS and PROCARDIS. SGCD SNPs in WGHS were not imputable due to low minor allele frequency. Beta-coefficients are given per copy of minor allele, assuming an additive model of inheritance, adjusted for age (and gender in PROCARDIS). rsq: imputation quality measure; MAF: minor allele frequency * Due to low MAF, homozygous and heterozygous carriers of the minor allele were pooled, hence the beta-coefficient is given for the presence of the minor allele. (0.03 MB DOC) [file pone.0014529.s007.doc]

|  |  | **WGHS (n=23,170)** | | | | | **PROCARDIS (n=3,078)** | | | | |
| --- | --- | --- | --- | --- | --- | --- | --- | --- | --- | --- | --- |
|  | **rsq** | **minor allele** | **major allele** | **MAF** | **beta (SE)** | **p** | **minor**  **allele** | **major**  **allele** | **MAF** | **beta (SE)** | **p** |
| SGCD |  |  |  |  |  |  |  |  |  |  |  |
| **rs10071215** | - | - | - | - | - | - | T | C | 0.011 | 0.0599* (0.0544) | 0.2799 |
| **rs6877118** | - | - | - | - | - | - | A | G | 0.014 | 0.0627* (0.0464) | 0.1764 |
| **Fibulin-5** |  |  |  |  |  |  |  |  |  |  |  |
| **rs2246416** | 0.997 | G | A | 0.30257 | -0.00389 (0.00473) | 0.41 | G | A | 0.304 | 0.0114 (0.0121) | 0.3470 |
| **rs3783937** | 0.998 | T | C | 0.24152 | -0.00257 (0.00509) | 0.61 | T | C | 0.238 | -0.0033 (0.0127) | 0.7947 |
